# Dissecting the mechanism of Yuzhi Zhixue granule on ovulatory dysfunctional uterine bleeding by network pharmacology and molecular docking

**DOI:** 10.1186/s13020-020-00392-0

**Published:** 2020-10-23

**Authors:** Jialin Li, Hua Luo, Xinkui Liu, Jingyuan Zhang, Wei Zhou, Siyu Guo, Xiuping Chen, Yingying Liu, Shanshan Jia, Haojia Wang, Bingbing Li, Guoliang Cheng, Jiarui Wu

**Affiliations:** 1 Department of Clinical Chinese Pharmacy, School of Chinese Materia Medica, Beijing University of Chinese Medicine, No. 11 of North Three-ring East Road, Chao Yang District, Beijing, 100102 China; 2 Institute of Chinese Medical Sciences, State Key Laboratory of Quality Research in Chinese Medicine, University of Macau, Macao, China; 3 State Key Laboratory of Generic Manufacture Technology of Chinese Traditional Medicine, Linyi, 276000 China

**Keywords:** Yuzhi zhixue granule, Ovulatory Dysfunctional Uterine Bleeding, Network pharmacology, Molecular Docking, Mechanisms

## Abstract

**Background:**

Yuzhi Zhixue Granule (YZG) is a traditional Chinese patent medicine for treating excessive menstrual flow caused by ovulatory dysfunctional uterine bleeding (ODUB) accompanied by heat syndrome. However, the underlying molecular mechanisms, potential targets, and active ingredients of this prescription are still unknown. Therefore, it is imperative to explore the molecular mechanism of YZG.

**Methods:**

The active compounds in YZG were screened by the Traditional Chinese Medicine Systems Pharmacology Database and Analysis Platform (TCMSP). The putative targets of YZG were collected via TCMSP and Search Tool for Interacting Chemicals (STITCH) databases. The Therapeutic Target Database (TTD) and Pharmacogenomics Knowledgebase (PharmGKB) databases were used to identify the therapeutic targets of ODUB. A protein–protein interaction (PPI) network containing both the putative targets of YZG and known therapeutic targets of ODUB was built. Furthermore, bioinformatics resources from the database for annotation, visualization and integrated discovery (DAVID) were utilized for Gene Ontology (GO) and Kyoto Encyclopedia of Genes and Genomes (KEGG) enrichment analyses. Finally, molecular docking was performed to verify the binding effect between the YZG screened compounds and potential therapeutic target molecules.

**Results:**

The study employed a network pharmacology method, mainly containing target prediction, network construction, functional enrichment analysis, and molecular docking to systematically research the mechanisms of YZG in treating ODUB. The putative targets of YZG that treat ODUB mainly involved PTGS1, PTGS2, ALOX5, CASP3, LTA4H, F7 and F10. The functional enrichment analysis suggested that the produced therapeutic effect of YZG against ODUB is mediated by synergistical regulation of several biological pathways, including apoptosis arachidonic acid (AA) metabolism, serotonergic synapse, complement and coagulation cascades and C-type lectin receptor signaling pathways. Molecular docking simulation revealed good binding affinity of the seven putative targets with the corresponding compounds.

**Conclusion:**

This novel and scientific network pharmacology-based study holistically elucidated the basic pharmacological effects and the underlying mechanisms of YZG in the treatment of ODUB.

## Background

Ovulatory dysfunctional uterine bleeding (ODUB), mainly occurring in women of childbearing age, is a common gynecological disease worldwide. Clinically, patients often have symptoms that include abnormal increases in menstrual flow, prolonged menstruation, dull pain in the lower abdomen, and a dark brown menstrual blood color. Clinically, the main causes of ODUB are luteal dysplasia and luteal atrophy, which lead to irregular shedding of the endometrium and prolonged menstruation. Sometimes, patients may have continuous bleeding before or after menstruation, and large menstrual volume [1–4]. ODUB is harmful to patients' quality of life and physical and mental health. If the patient is not treated effectively on time, which will lead to the continued development of the disease, it may also pose a threat to the patient's life and safety. Previous studies have revealed that Western medicines such as progesterone can improve the clinical symptoms of patients. However, Western medicines have the disadvantages of long use cycles and easy relapse after withdrawal. Some patients may also have serious gastrointestinal adverse reactions during the treatment period. Therefore, it is urgent to develop more effective and safer alternatives for curing ODUB [5–7].

Traditional Chinese medicine (TCM) has been widely used for the treatment and prevention of various diseases, especially in Asian countries, including China, Japan, and South Korea [8]. In TCM, ODUB falls into the category of "collapse leakage", which means that menstrual blood is constantly being exposed or flowing. Deficiency of the spleen and kidney and Chong-Ren debility are all closely related to collapse leakage and involve the heart, liver and kidney. Collapse leakage is divided into cold, heat, deficiency and empirical symptoms, but heat syndrome accompanied by stasis is more common in clinical trials. The belief in TCM is that the key to treating collapse leakage lies in strengthening the spleen and kidney, clearing heat and regulating the menstrual cycle as well as emphasizing the treatment based on syndrome differentiation and holistic treatment. Many studies have confirmed that TCM exerts superb clinical effects in treating dysfunction, with treatment characteristics of long-lasting and slight side effects [9, 10].

YZG is composed of 17 kinds of TCM, including *Sanguisorba officinalis* L.(Diyu), *Gardenia jasminoides* Ellis (Zhizi), *Cirsium japonicum* Fisch.ex DC. (Daji), *Sophora japonica* L.(Huaihua), *Rehmannia glutinosa* Libosch.(Dihuang), *Paeonia suffruticosa* Andr. (Mudanpi), *Rubia cordifolia* L.(Qiancao), *Polygonum bistorta* L.(Quanshen), *Scutellaria baicalensis* Georgi (Huangqin), *Paeonia lactiflora* Pall. (Baishao), and *Angelicasinensis*(Oliv.) Diels (Danggui), which have the functions of clearing heat, cooling blood, and stopping bleeding. Its main traditional function is to treat ODUB caused by heavy menstruation and syndromes belonging to blood fever in TCM. Patients can be accompanied by dry mouth and upset, red tongue and yellow skin. In the YZG formula, Diyu is used for cooling blood and hemostasis, and Zhizi can cool blood, clear away the heat syndrome and agitation. Diyu and Zhizi are concerned as the “Monarch” medicine in the prescription. Daji, *Platycladus orientalis*(L.)Franco (Cebaiye), Huaihua, Quanshen, Huangqin and *Eclipta prostrate* L. (Mohanlian) are mainly used to cool blood and stop bleeding, which can enhance the ability of “Monarch” medicine on cooling blood and stopping bleeding, alleviating bleeding with heat syndrome. They are regarded as the “Minister” medicine in the prescription. Qiancao, *Agrimonia pilosa* Ledeb.(Xianhecao) can treat blood leakage; Typha angustifolia L.(Puhuang) is good for removing blood stasis and stopping bleeding; Dihuang and Mudanpi are good at clearing heat, cooling blood. These Traditional Chinese medicine can cooperate with “Monarch” medicine and “Minister” medicine to treat ODUB with fever syndrome. They can be identified as “Assistant” medicine. The Baishao and Danggui that can nourish the blood and kidney play a role in“Guide”medicine. It can reconcile the properties of various drugs in the prescription and enhance the nourishing effect. The combination of all medicines can not only strengthen the effect of hemostasis, regulate menstruation and clearing away heat, but also nourish yin and blood, preventing physical weakness. ODUB belongs to the premenstrual and prolonged periods in TCM. TCM theory indicates that the deficiency of Yin fluid and endogenous heat will show menstrual blood without containment, further inducing hypermenorrhea and menostaxis. YZG has the function of nourishing Yin and clearing heat, cooling blood and hemostasis. Hence, it can be used to treat ODUB, which will achieve an excellent effect [11, 12]. However, the molecular mechanism of YZG in the treatment of ODUB is still unclear.

**Fig. 1:**
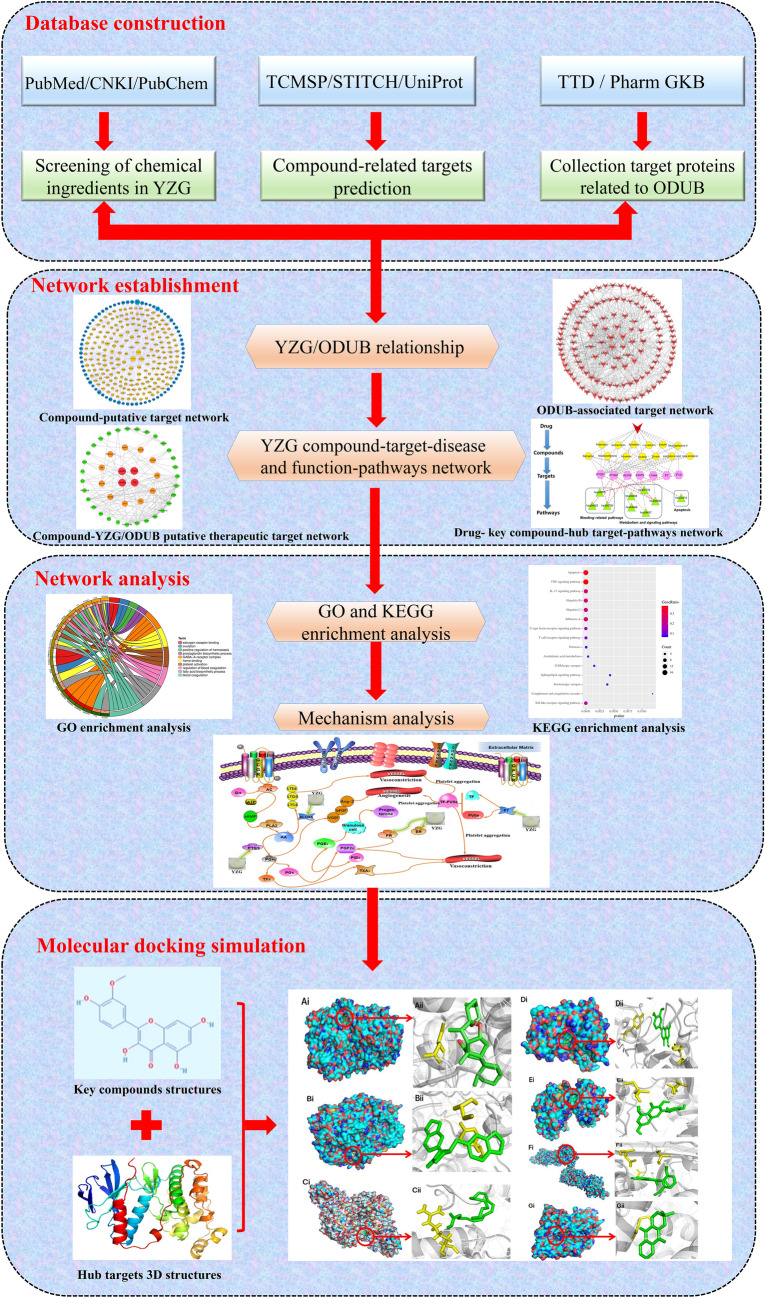
Workflow for Yuzhi Zhixue Granule (YZG) in the treatment of ODUB

Network pharmacology is a novel discipline based on network theory and systems biology principles, which is deemed as an effective tool for systematically revealing complex network relationships [13, 14]. TCM and its formulas possess the characteristics of synergistic effects of multi-component, multi-target, and multi-pathway. Network pharmacology can reveal the complex overall biological network relationships among drugs, ingredients, targets, and diseases, providing a new perspective for analyzing and predicting the pharmacological mechanism of drugs [15]. Therefore, a high degree of consistency is exerted in network pharmacology and TCM at the holistic and systemic level, granting a new opportunity for in-depth research of TCM compounds [16]. Because the mechanism of YZG cannot be fully explained by the individual TCM ingredients alone, the network pharmacology method was applied to illuminate the hemostatic molecular mechanism of this Chinese herbal prescription. This study aims to preliminarily shed light on the potential mechanism of YZG in the treatment of ODUB through network pharmacology and molecular docking. The detailed workflow is shown in Fig. 1.

## Methods

### Screening the active compounds of YZG

In this research, all the chemical components of YZG were identified from the traditional Chinese medicine system pharmacology database and analysis platform (TCMSP, https://tcmspw.com/tcmsp.php) [17]. TCMSP, a unique system pharmacology platform devised for Chinese herbal medicines, can provide information on the chemical substances of TCM, targets, and pharmacokinetic properties of natural compounds. The screening threshold of the oral bioavailability (OB) was set to ≥ 30%, and the drug-likeness (DL) was set to ≥ 0.18 to screen the effective active components of YZG [18]. The active components of YZG were selected from STITCH database with high confidence (0.700) as threshold. A total of 161 chemical ingredients of YZG were retrieved from the TCMSP. After deleting duplicate data and compounds without structural information, 121 herbal compounds were ultimately collected [19, 20].

### The prediction of putative targets of the collected compounds

To identify the compound targets of YZG, we chose two widely used databases: STITCH and TCMSP. STITCH (https://stitch.embl.de/) is a database of known and predicted interactions between compounds and protein, and is based on text mining and molecular docking techniques [21]. STITCH has been used to explore the potential active components and to explain the molecular mechanism of TCM. All the collected compounds entries into the STITCH database were imported one by one, the species were limited to human species, and the target gene names of compounds were collected. In addition, the target proteins of all compounds obtained from the TCMSP database were imported into the UniProt database to obtain the gene names corresponding to the target proteins. Subsequently, we acquired all corresponding targets after removing repetitive targets. The UniProt database (https://www.UniProt.org/), which contains a large number of protein sequences and detailed annotated information, was used to find the gene name corresponding to the proteins; only human targets were reserved [22].

### Construction of the compound-target network

All the compounds that possess targets are were numbered according to their molecular ID number, and a series of numbers with their relevant targets are were imported into the Cytoscape 3.6.1 software (https://www.cytoscape.org/) to construct a compound-predicted target network map. Cytoscape is a bioinformatics analysis software that visualizes biological pathways and intermolecular interaction networks. It provides a basic set of data integration, analysis and visualization capabilities for complex network analyses [23, 24].

### Collection of target proteins for related diseases

The therapeutic target database (TTD, https://db.idrblab.org/ttd/), is a database that provides information about nucleic acid targets and the therapeutic effects of proteins [25]. The pharmacogenomics knowledgebase (PharmGKB, https://www.pharmgkb.org/) is a database that collects complete genotypic and phenotypic information related to the pharmacogenomics and systematically categorizes this information [26]. We used these two databases to collect target genes related to menstruation by searching for with keywords “hemorrhage”, “dysmenorrhea” “endometriosis”, “bleeding disorder”, “excessive bleeding”, “pain” and “inflammation” and then set up a data set for menstruation with multiple correlations.

### Constructing a protein–protein interaction (PPI) network for the disease target

All the targets associated with excessive menstruation were input into the STRING (https://string-db.org/) database, with the species limited to “Homo sapiens” and confidence scores higher than 0.7 [27]. The STRING database aims to collect, score, and integrate all publicly available sources of PPI information and to complement these with computational predictions. Its goal is to achieve a comprehensive and objective global network, including direct (physical) as well as indirect (functional) interactions. STRING defines PPIs with confidence ranges for data scores (low: < 0.4; medium: 0.4 to 0.7; and high: > 0.7). The size of the nodes is directly proportional to the degree of the nodes, and each edge represents the interaction between the compound molecules and targets.

### Collection of overlapping targets

By using the merge function in the Cytoscape software, we matched the prediction of the targets of the YZG active ingredients and the retrieval of the related targets of abnormal menstruation. Then, the overlapping targets were chosen as the related targets of YZG in treating ODUB. The targets were then processed by the STRING database to draw the PPI data. Then, a potential key target network for the YZG treatment of ODUB was constructed by Cytoscape, and the potential targets of the network were systematically analyzed [28, 29].

### Screening for hub genes

We adopted the plug-in CytoHubba in Cytoscape software to analyze the data in the PPI network of overlapping targets by the bottleneck algorithm, aiming to find hub–bottleneck genes. Hub–bottleneck genes are considered highly central proteins that connect several complexes, which are more likely to be part of signal transduction pathways. The parameters were set as the top = 15 and were ranked by the hub–bottleneck [30].

### Gene Ontology (GO) and Kyoto Encyclopedia of Genes and Genomes (KEGG) pathway enrichment analyses

To shed light on the potential mechanism of YZG and its effects on ODUB, the functional pathways of YZG related to excessive menstruation diseases were analyzed using the KEGG pathway and GO enrichment analyses based upon the database for annotation, visualization and integrated discovery (DAVID) version 6.8 (https://david.ncifcrf.gov/). DAVID, a high-throughput and integrated data-mining environment, analyses gene lists derived from high-throughput genomic experiments. P-value < 0.01 and FDR < 0.05 were considered as the criteria for difference screening [31, 32].

### Molecular docking simulation

The molecular docking software AutoDock was used to validate the network pharmacology screening results by docking the active compound with the proteins. Molecular docking refers to placing small-molecule ligands on the binding region of large-molecule receptors by computer simulation and then calculating the physical and chemical parameters to predict the binding affinity between the two. We applied AutoDockTools 1.5.6 to process ligands and receptors and used AutoDock Vina 1.1.2 for molecular docking and analysis of docking results [33, 34]. Before docking, the energy of the ligands and acceptors needs to be minimized, the water molecules small molecules of acceptors (PDB files) need to be deleted, polar hydrogen atoms need to be added, and the charge and magnetic field need to be added. The results were visualized by PyMOL, and the hydrogen bonds and their binding sites were observed and analyzed. The docking energy value was determined by the consistency score function of the ligand-receptor affinity [35]. The purpose of this study was to examine the binding free energy of the compound with the corresponding target to determine the affinity between them. The smaller the binding free energy required for docking is, the greater the affinity.

## Results

### Screening active compounds and potential targets

The compounds were collected in the TCMSP and screened by OB ≥ 30% and DL ≥ 0.18. A total of 115 compounds from YZG were screened, and among which, 72 compounds that had targets were retained. Of these compounds, 23 were from Huangqin, 12 from Qiancao, 11 from Zhizi, 9 from Mohanlian, 7 from Baishao, 7 from Cebaiye, 6 from Diyu, 6 from Mudanpi, 6 from Puhuang, 6 from Daji, 5 from Huaihua, 5 from Quanshen, 4 from Xianhecao, 2 from Dihuang, and 2 from Danggui. Compounds such as ( +)catechins, sitosterol, kaempferol and quercetin exist in a variety of TCM in YZG, suggesting that these compounds may be necessary for therapeutic effects.

### Compound-putative target network construction

**Fig. 2:**
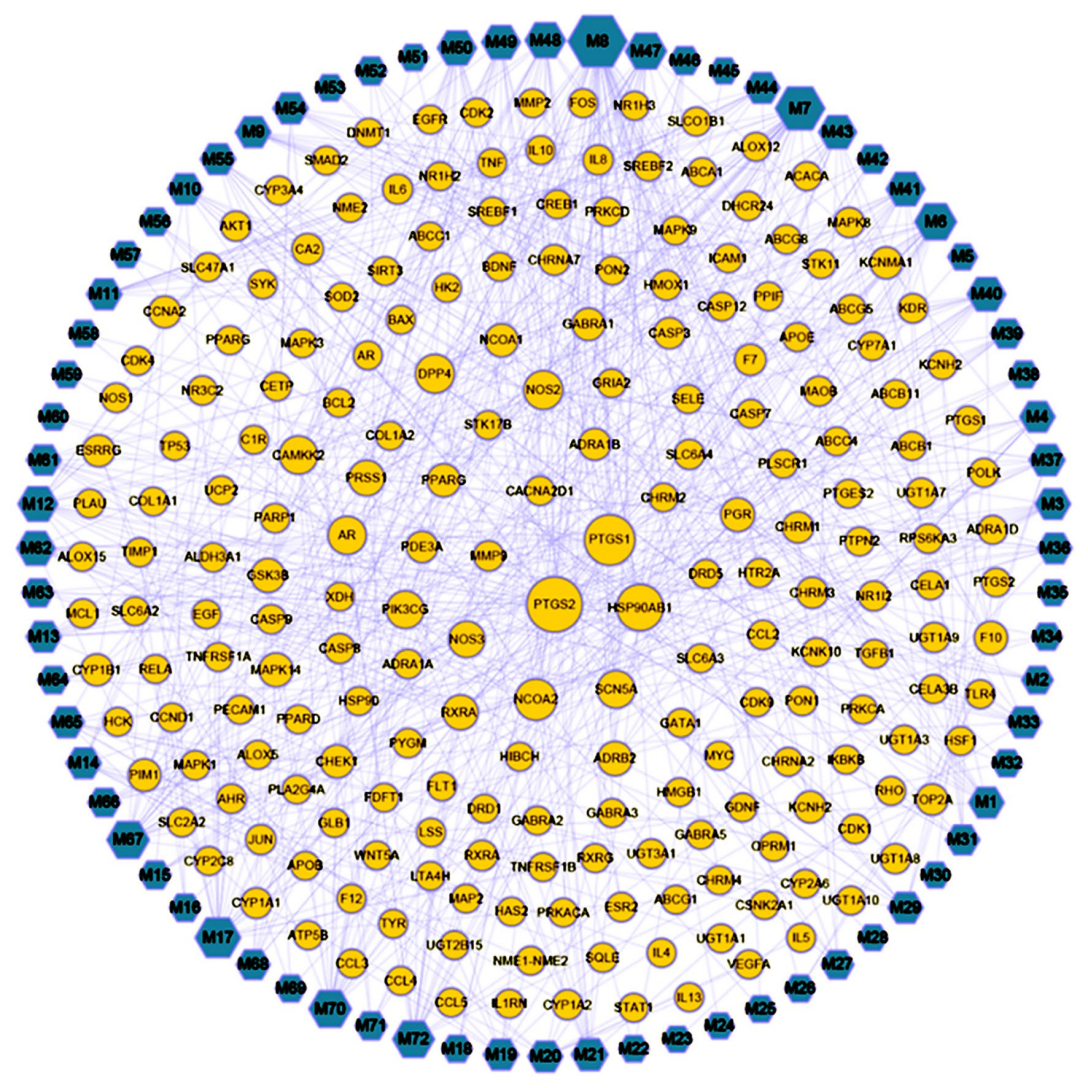
Compound-target network of YZG. (Blue circles represent the compounds, orange circles represent the targets, and edges represent interactions between compounds and targets)

We used Cytoscape to construct a compound–compound target network that comprised 285 nodes (72 compound nodes and 213 target nodes) and 654 edges. The blue nodes represent the compound molecules, and the orange nodes represent the compound targets. Each edge represents the interaction between the compound and its target (Fig. 2). In the network, the degree of a node indicates the number of routes it takes to connect to other nodes. According to the topological properties of the network, nodes with a larger degree were screened for further analysis. These nodes, together with more connected compounds or targets, play a pivotal role in the network and may be key compounds or targets. In this network, there is a phenomenon in which a compound corresponds to multiple targets, and several compounds share a common target simultaneously. This reflects the YZG mechanism that comprises multiple components and targets, which is in accordance with the characteristics of many TCM prescriptions and drugs that have a curative effect on the treatment. The number of targets of 47.2% compounds was 10 or more, and the number of targets for seven compounds was 20 or higher. Because AA can act on a variety of targets, it may be the crucial compound of YZG that exerts a hemostatic effect. Regarding the targets, the top three were PTGS2, PTGS1 and HSP90AB1, which can interact with 46, 35, and 27 compounds, respectively. These three targets may be essential for the actions of the compounds. According to the degree of the compound, we finally chose the 13 more important compounds, and the details were shown in Table 1.

**Table 1:** Information on active components

No	CAS	Compound	Degree	Structure	Herb
1	7771-44-0	Arachidonic acid	26		Puhuang
2	480-19-3	Isorhamnetin	32		Puhuang Huaihua
3	83-46-5	Beta-sitosterol	47		Cebaiye Puhuang Danggui Quanshen Diyu Zhizi
4	1447-88-7	Dinatin	10		Daji
5	26,543-89-5	Hinokinin	10		Cebaiye
6	3570-62-5	Moslosooflavone	21		Huangqin
7	70,028-59-0	Rivularin	20		Huangqin
8	19,103-54-9	Salvigenin	15		Huangqin
9	15,297-92-4	Xyloidone	21		Qiancao
10	2284–31-3	Pratensein	16		Mohanlian
11	55,084-08-7	Skullcapflavone II	14		Huangqin
12	491-70-3	Luteolin	10		Xianhecao
13	88,191-48-4	( +)-catechin	17		Baishao Xianhecao Mudanpi Mohanlian

### Construction of the PPI network for the corresponding disease

**Fig. 3:**
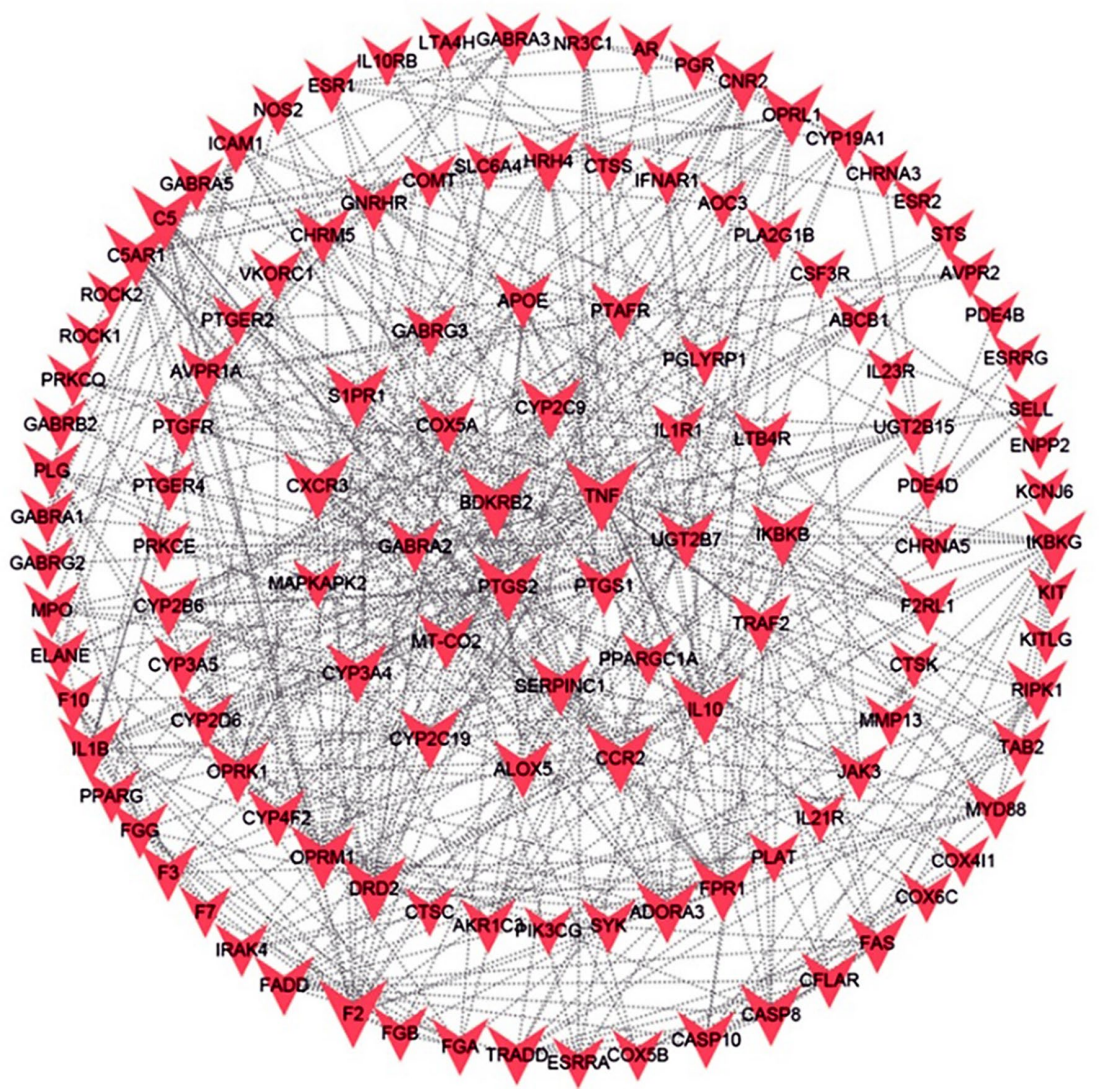
PPI network with the targets for ODUB. (The red Vs represent the target of the disease, and each edge represents the interaction between the targets)

A total of 100 targets of ODUB-related diseases were retrieved from the TTD and PharmGKB databases, and the genes were entered into the STRING database for obtaining a protein interaction, and finally, 126 disease-related targets were obtained from STRING database (see Fig. 3).Three topological features of each node in the network were calculated to find the major nodes. We selected the targets with the top five grades of degree, betweenness and closeness as the key disease targets, which may be studied in further research. The 5 nodes with degree ≥ 18 were TNF, IL10, BDKRB2, F3, and PTGS2; the 5 nodes with betweenness ≥ 0.06 were TNF, PTGS2, IL10, SYK, and CXCR; and the 5 nodes with closeness = 1 were GABRA1, GABRB2, GABRA5, GABRA2, and GABRA3, and these were considered genes essential to the development of ODUB.

### PPI network of targets for YZG against ODUB

**Fig. 4:**
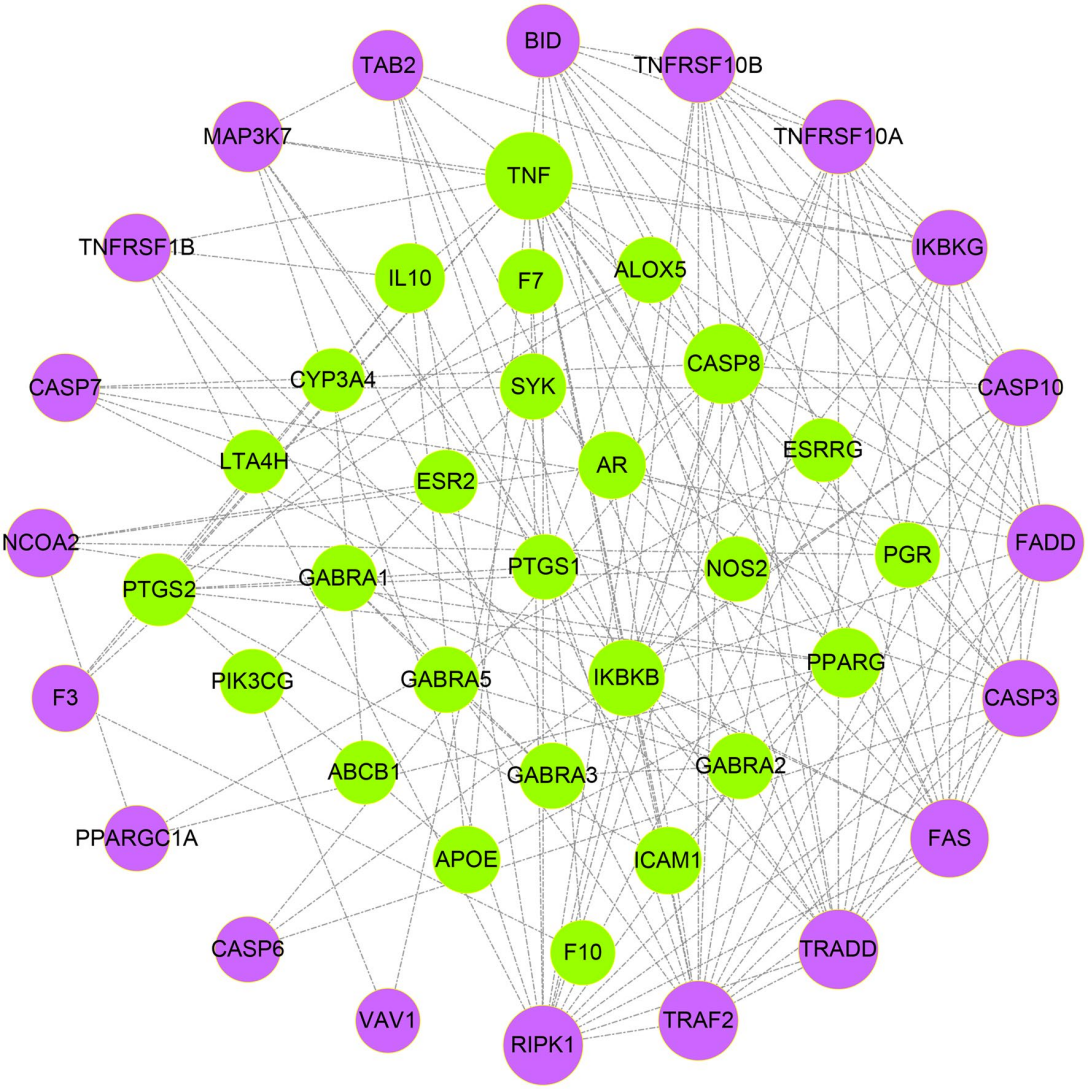
The PPI network of overlapping targets of compound-disease. (Green targets represent primary proteins, and purple targets represent secondary proteins)

By merging compound-target and PPI networks for ODUB, we obtained 29 overlapping protein targets, which can be considered the potential therapeutic targets. Using these overlapping targets, we searched the STRING database and found, and a total of 17 interacting secondary proteins that were associated with potential therapeutic targets. As a result, the network was composed of 46 nodes and 155 edges (Fig. 4).

### Hub genes

**Fig. 5:**
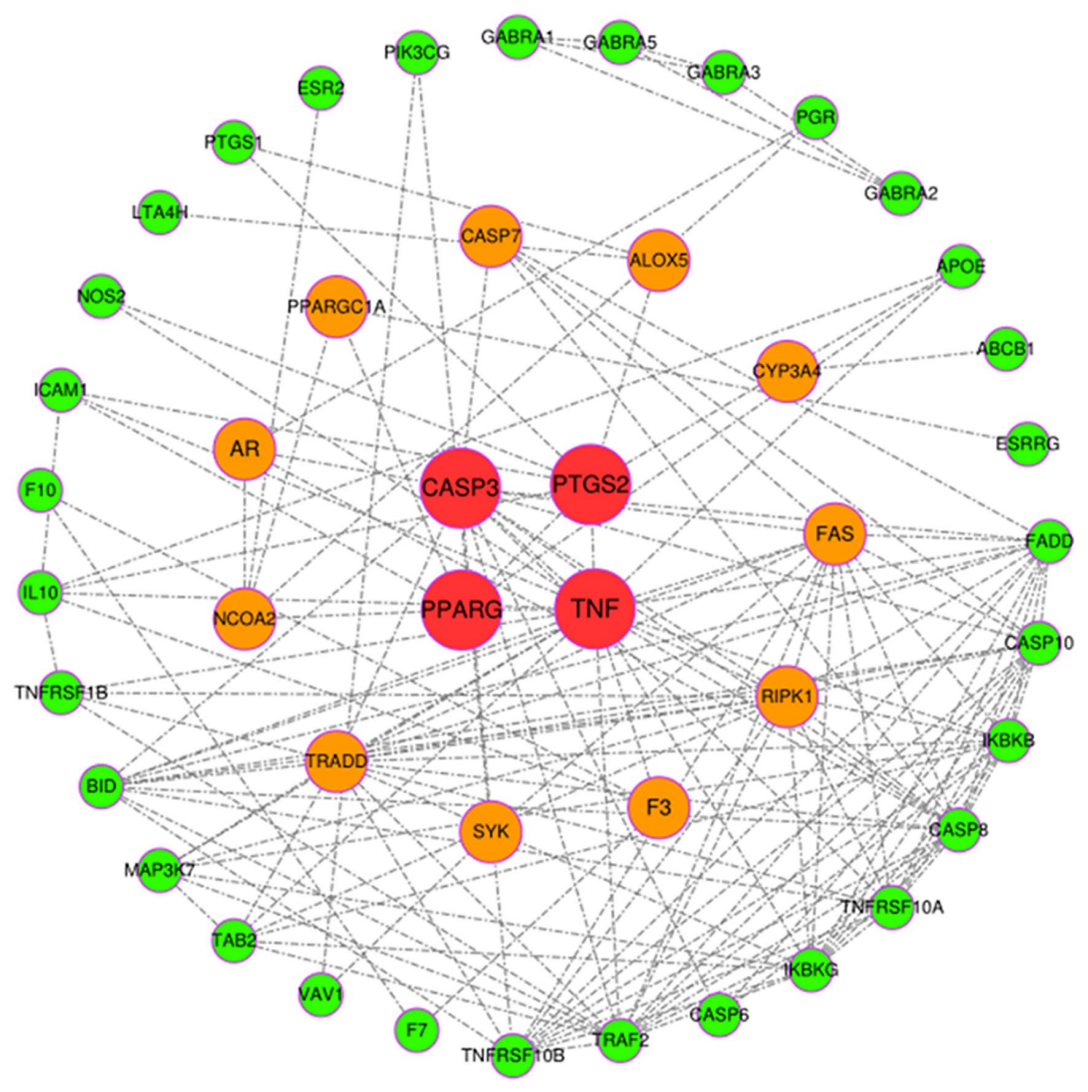
The hub genes of PPI network of overlapping targets. (The red targets refer to hub genes with scores greater than 5, orange targets represent genes with scores greater than 1 and less than 5, and green targets represent genes with lower scores)

We applied the bottleneck algorithm of the plug-in CytoHubba in Cytoscape software and found a total of 15 hub–bottleneck genes. There were four genes, PTGS2, CASP3, TNF, and PPARG, with a score of ≥ 5 and accompanied by red dots. The score of 11 genes, namely, ALOX5, CASP7, CYP3A4, FAS, F3, SYK, RIPK1, TRADD, NCOA2, AR, and PPARGC1A, was ≥ 1, and these were represented by orange dots (Fig. 5). These may be key genes that play a vital role in the pathway, implicating that we should focus on their respective pathways and conduct a detailed analysis.

### GO function and KEGG pathway enrichment analyses

**Fig. 6:**
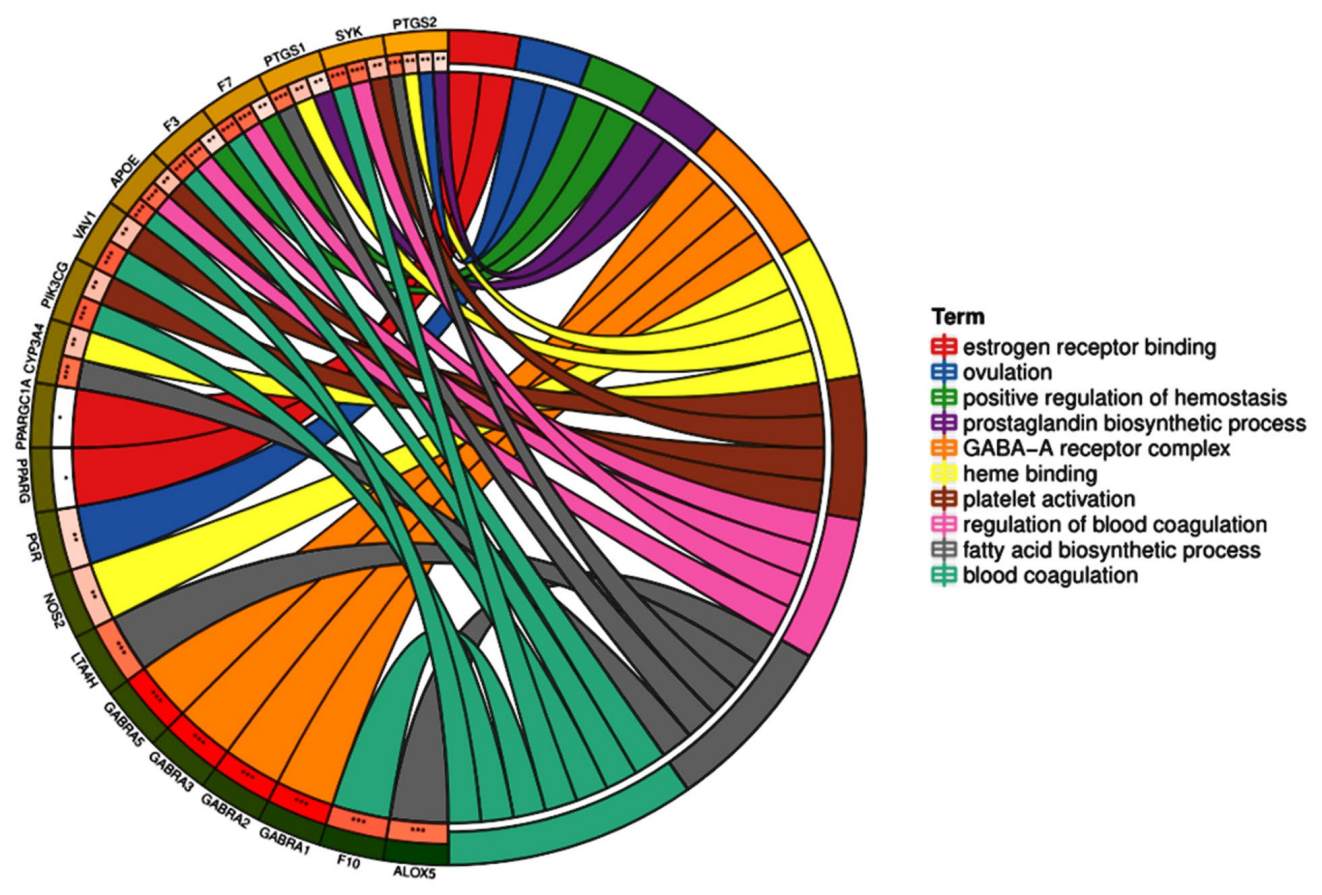
GO enrichment analysis of major proteins. (Term is on the right side of the circle, and the gene is on the left side. The corresponding color of the gene ribbon is consistent with the color of Term, indicating that this gene is enriched in this term)

**Fig. 7:**
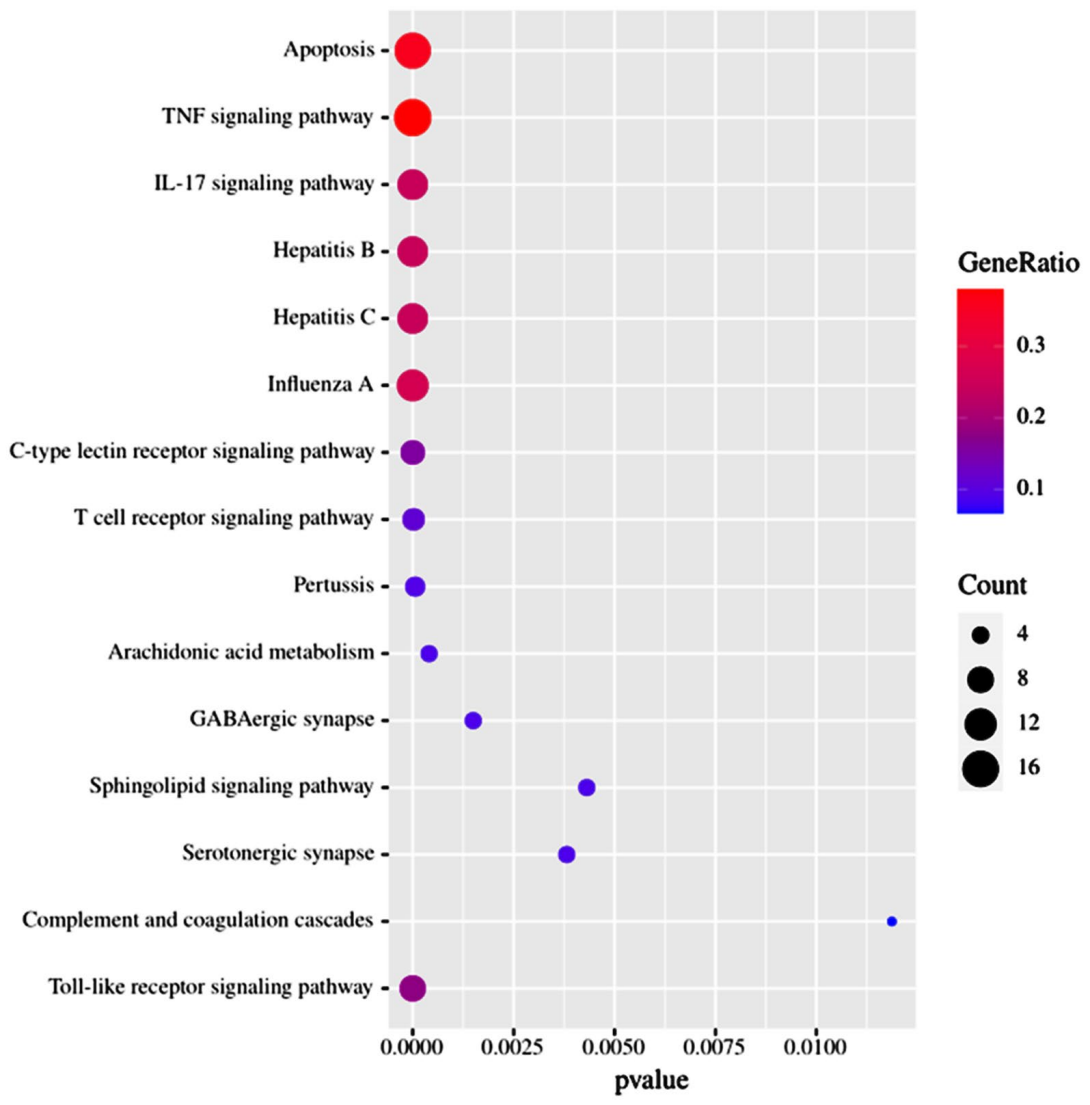
KEGG enrichment analysis of major proteins. The y-axis shows significantly enriched KEGG pathways, and x-axis shows the rich factor. Rich factor represents the ratio of the number of target genes belonging to the pathway to the number of all annotated genes located in the pathway. The larger rich factor stands for the higher level of enrichment. The size of the dot denotes the number of target genes in the pathway, and the color shade of the dot indicates the different P-value range

**Fig. 8:**
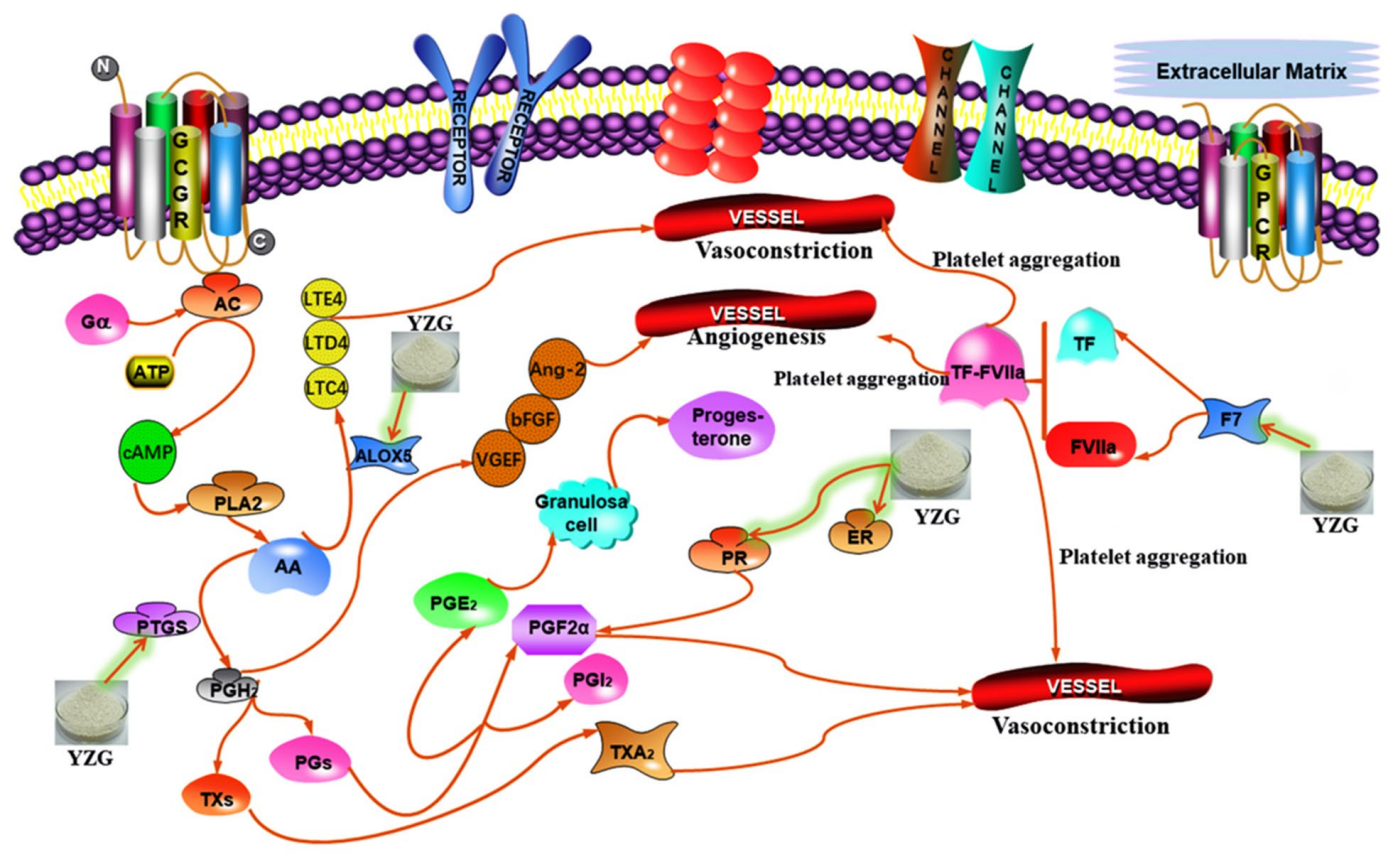
Hemostatic routes influenced by major putative targets of YZG

To elucidate the biological functions of these major hubs, we analyzed the candidate targets by performing a GO enrichment analysis, and the top 29 significant GO entries (P-value < 0.05) were chosen according to the P-value, as shown in Fig. 6. The results indicated that the major hubs were significantly involved in multiple biological processes, primarily including inflammatory response, blood coagulation, hemostasis, coagulation, regulation of blood coagulation, regulation of hemostasis, fever generation, regulation of heat generation, platelet activation, ovulation, positive regulation of hemostasis, positive regulation of coagulation. The results of the cellular component and molecular function analyses showed that important genes exist in the cell body, GABA-ergic synapses, whose functions at the molecular level mainly included heme binding and estrogen receptor binding. The processes of blood coagulation, hemostasis, positive regulation of hemostasis, ovulation, positive regulation of coagulation, response to gonadotropin, heme binding, and estrogen receptor binding are related to abnormal menstrual bleeding in women. Thus, we speculated that YZG exerted its pharmacological effects on ODUB by simultaneously involving these biological processes and molecular functions. With the help of DAVID bioinformatics resources, 70 KEGG pathways with P-values ≤ 0.05 were also collected and analyzed. The top 15 crucially significant pathways were selected for further study and are displayed in the advanced bubble diagram (Fig. 7). These pathways were associated with the TNF signaling pathway (hsa04668), IL-17 signaling pathway (hsa04657), hepatitis B (hsa05161), hepatitis C (hsa05160), influenza A (hsa05164), C-type lectin receptor signaling pathway (hsa04625), T-cell receptor signaling pathway (hsa04660), Pertussis (hsa05133), AA metabolism (hsa00590), GABA-ergic synapse (hsa04727), sphingolipid signaling pathway (hsa04071), serotonergic synapse (hsa04726), complement and coagulation cascades (hsa04610), Toll-like receptor signaling pathway (hsa04620), and apoptosis (hsa04210). Detailed information about the top 15 significant pathways was supplemented in Supplementary Fig. 7. It is worthy of note that the hub genes we previously found, was mostly enriched the AA metabolism, serotonergic synapses, complement and coagulation cascades, C-type lectin receptor signaling pathway, apoptosis, TNF signaling pathway, and IL-17 signaling pathway, which suggested that the compounds in YZG may treat ODUB through the above pathways by acting on related targets. Importantly, by combining the obtained information of compound-target and hub genes, we found that PTGS1, PTGS2, ALOX5, CASP3, LTA4H, F7 and F10 were considered key targets because they also exist in important pathways related to bleeding (Fig. 8).

### Molecular docking verification

**Fig. 9:**
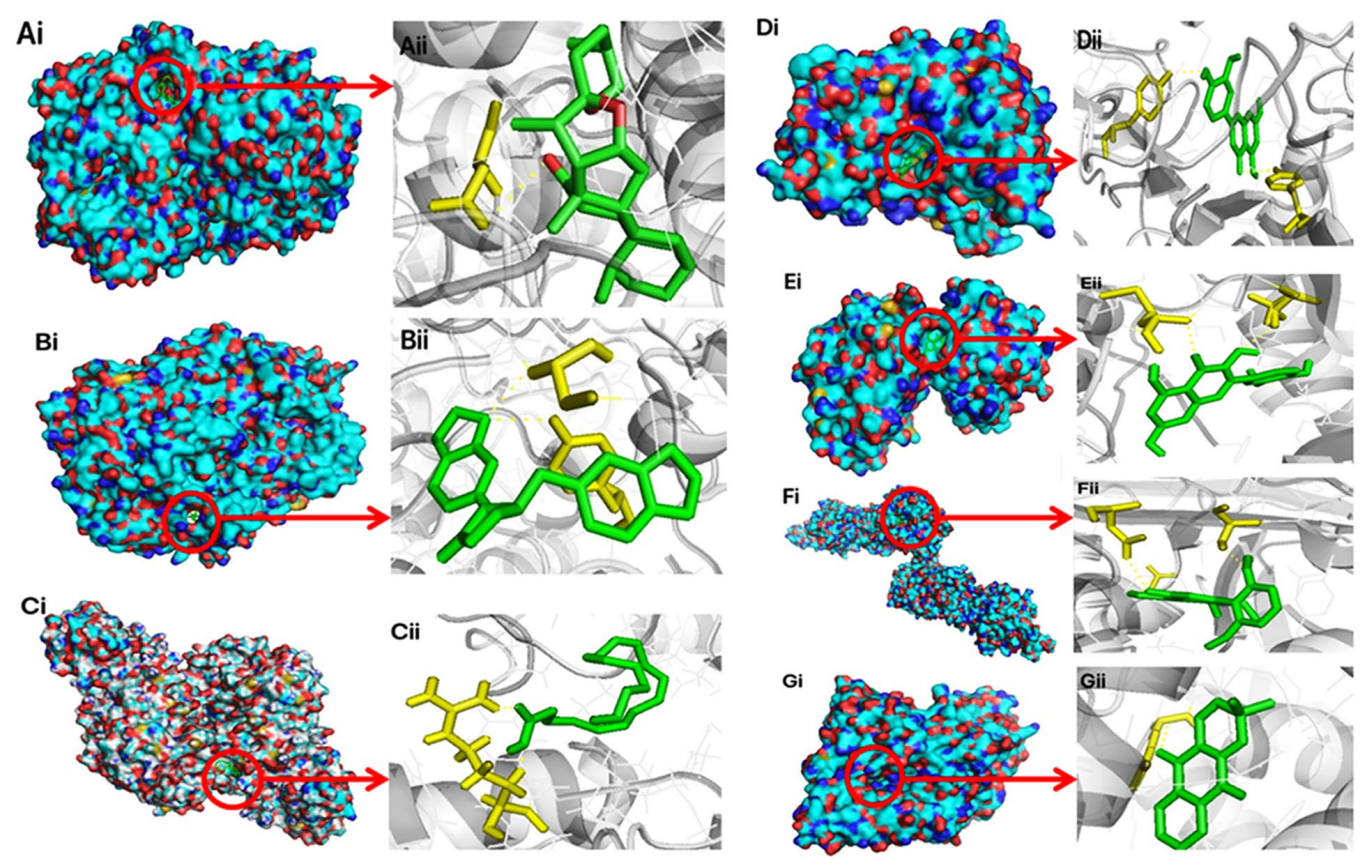
The detailed target-compound interactions of the docking simulation. (The red circle indicates the docking state between small molecule compound and large molecule protein, and the detailed docking condition on the right side can be observed)

Through molecular docking experiments, the numerical results of the binding energies from Vina docking were collected. The docking details are shown in Table 2, and the binding energy values of the 13 important compounds in YZG with their main targets are all less than − 5 kcal/mol (20.9 kJ/mol), which suggests that the binding is significant; the less energy that is required, the more stable the binding. We selected the targets that bind to the most stable compound to display the 3D results, which included PTGS1-β-sitosterol (affinity = − 10.6 kcal/mol), PTGS2-Hinokinin (affinity = − 9.8 kcal/mol), ALOX5-AA (affinity = − 5.1 kcal/mol), CASP3-luteolin (affinity = − 7.4 kcal/mol), F7-isorhamnetin (affinity = − 8.4 kcal/mol), F10-rivularin (affinity = − 8.0 kcal/mol), and LTA4H-xyloidone (affinity = − 8.5 kcal/mol). In Fig. 9, the red circle represents a small-molecule compound, and each small-molecule compound is bound to a large-molecule protein. The stick graph on the right describes the specific form of this interaction. The yellow dashed line in the figure represents hydrogen bonding; there are fewer hydrophobic residues around the compound, and these residues are mainly bound to the target by electrostatic interactions (hydrogen bonding).

**Table 2:** Information on molecular docking

No	Proteins	PDB ID	Protein structure	Test compounds	Affinity (kcal/mol)
1	PTGS1	3N8W		Arachidonic acid ( +)-catechin Beta-sitosterol Dinatin Hinokinin Isorhamnetin Moslosooflavone Pratensein Rivularin Salvigenin SkullcapflavoneII Xyloidone	− 5.3 − 7.7 − 10.6 − 8.8 − 9.5 − 9.5 − 8.5 − 9.2 − 8.4 − 8.6 − 8.1 − 8.8
2	PTGS2	5F19		Arachidonic acid SkullcapflavoneII Salvigenin Rivularin Pratensein Moslosooflavone Isorhamnetin Hinokinin Dinatin Beta-sitosterol ( +)-catechin Xyloidone	− 5.7 − 9.4 − 9.2 − 9.5 − 9.4 − 9.5 − 9.3 − 9.8 − 9.6 − 9.4 − 9.2 − 9.1
3	ALOX5	3O8Y		Arachidonic acid	− 5.1
4	CASP3	3GJR3GJR		Luteolin Arachidonic acid	− 7.4 − 6.2
5	F7	4EH8		SkullcapflavoneII Salvigenin Rivularin Isorhamnetin	− 7.8 − 8.0 − 8.0 − 8.4
6	F10	2GD4		SkullcapflavoneII Salvigenin Rivularin	− 7.5 − 7.8 − 8.0
7	LTA4H	3B7S		Xyloidone	− 8.5

### Drug-compound-target-pathway network construction

**Fig.10:**
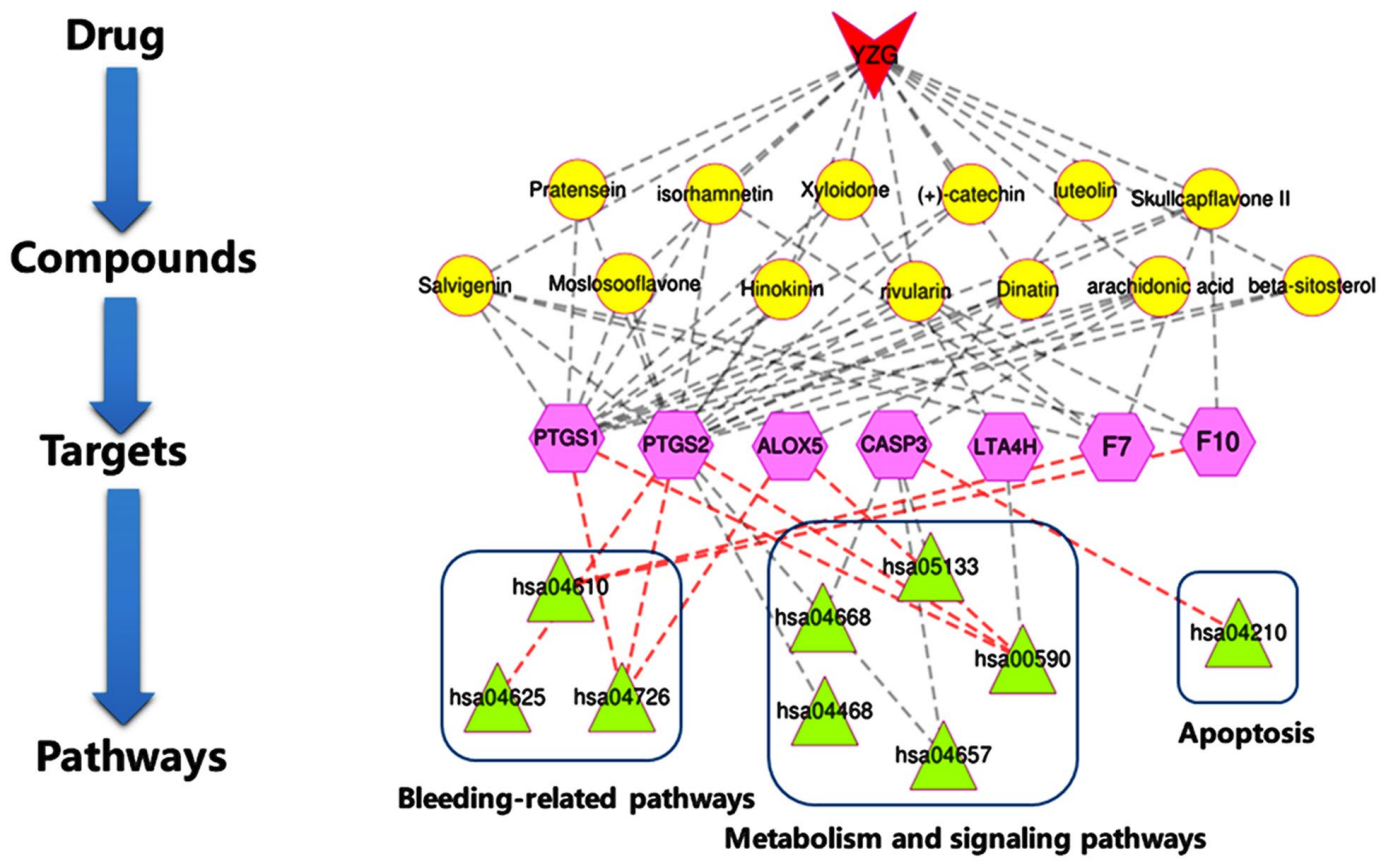
Drug-compound-target-pathway network. (The red Vs represent the drug YZG. Yellow circles represent the compounds. Pink regular hexagon targets represent compound/disease targets. Green triangles represent the predictive targets KEGG enrichment pathway)

To holistically explain the interaction mechanisms among the different drugs, compounds and main targets in YZG, a drug-compound-target-pathway network was constructed established based on Cytoscape software. Red indicates the drugs; yellow indicates the compounds; pink indicates the potential targets; and green indicates the pathways. The red lines are connected to the potential therapeutic pathway, which has the highest degree of value, its key targets and the components with the highest docking scores (Fig. 10). In YZG, flavonoids, coumarins, and organic acids are the compounds that are most closely related to crucial potential targets. Existing animal experiments have shown that flavonoids are the main active ingredients of *Platycladus orientalis *(L.), Franco (Cebaiye), *Cirsium japonicum* Fisch.ex DC. (Daji), *Cirsium setosum* (Willd.) MB. (Xiaoji) in cooling and hemostatic effects. The effective active ingredients of TCM for clearing heat and stopping bleeding also contain organic acids, phenols, and tannins. TCMs, including such as Cebaiye, Daji, Xiaoji, *Rubia cordifolia* L. (Qiancao), and *Sanguisorba officinalis* L. (Diyu) can achieve hemostasis by shortening bleeding and clotting times. In contrast, Qiancao are mainly used to increase the number of platelets and enhance their aggregation to ensure hemostasis [36–38]. However, the molecular mechanism of these compounds in treating uterine bleeding and heavy menstrual flow is unclear, which will be the focus of the discussion.

## Discussion

Unlike Western medicines, TCM treats diseases by regulating the overall bias of the body; hence, TCM often appears in the form of prescriptions containing many herbs and ingredients [39]. YZG has the functions of clearing heat, cooling blood, and stopping bleeding. The efficacy of this prescription can be explained by the theory of TCM compatibility. However, the molecular mechanisms underlying the effects of YZG are not clear. Hence, in the present study, a set of network pharmacology methods was used for predicting, elucidating, and confirming the potential mechanisms of action of YZG on ODUB by integrating target prediction, network construction, and molecular docking analyses. The analysis of the compound–compound target network and hub genes of the PPI network for intersecting targets revealed that PTGS2, PTGS1, CASP3, CASP7, TNF, ALOX5, F7, and F10 might be the key targets of YZG in ODUB. In this study, GO enrichment analysis showed that these targets were highly connected with the positive regulation of metabolic process, blood coagulation, regulation of hemostasis, regulation of heat generation, platelet activation, estrogen receptor binding, and prostaglandin biosynthetic process. Therefore, the results suggest that compounds in YZG mainly produce therapeutic effects by participating in these biological processes, cellular components, and molecular functions. The network analysis results revealed that YZG has the potential to influence a variety of biological pathways that play a crucial role in the pathogenesis of ODUB, including the AA metabolism, serotonergic synapse, C-type lectin receptor signaling pathway, apoptosis pathway, and complement and coagulation cascades.

Prostaglandin-endoperoxide synthase (PTGS), also known as cyclooxygenase, is the essential enzyme in prostaglandin biosynthesis. There are two isozymes of PTGS: constitutive PTGS1 and inducible PTGS2, which can catalyze AA to generate unstable prostaglandin G2 (PGG2) and prostaglandin H2 (PGH2), respectively; the latter is quickly converted to various prostaglandins (PGs) by different enzymes. Under the action of isomerase and synthetase, PGH2 produces the relatively stable prostaglandin E2 (PGE2) and prostaglandin F2α (PGF2α), while under the action of thromboxane synthase or prostaglandin synthetase, PGH2 produces thromboxane A2 (TXA2) and prostacyclin (PGI2). PTGS1 regulates angiogenesis in endothelial cells and is thought to be involved in cell–cell signaling and maintaining tissue homeostasis. PTGS2, a rate-limiting enzyme for AA to synthesize PGs, plays an important role in a series of reproductive processes involving ovulation, fertilization, implantation, and childbirth [40–42]. AA, which is one of the starting materials for PG biosynthesis, is an important and valuable compound in YZG [43]. Endometrial vasomotor dysfunction can lead to prolonged bleeding and increased menstrual flow. Many members in the prostaglandin family can coordinate to regulate vasomotor contraction. Among them, PGF2α is a vasoconstrictor that can promote microvascular contracture and thus stops bleeding. PGs can also promote angiogenesis by regulating several vascular endothelial growth factors, thereby promoting endometrial repair. Vascular endothelial growth factor (VEGF) is a member of a powerful group of cytokines that can produce multiple effects. It has specific selectivity for vascular endothelial cells and has a crucial effect on the development of endometrial blood vessels. VEGF binds to specific receptors on the surface of vascular endothelial cells, which proliferate vascular endothelial cells, enhance vascular permeability and vasodilation, and induce angiogenesis [44]. VEGF can provide an important matrix for the formation of vascular endothelium, making the endometrial vascular structure develop well and achieving the purpose of hemostasis. Basic fibroblast growth factor (bFGF), a member of the fibroblast growth factor family, can effectively promote mitosis of micro vascular endothelial cells and induce angiogenesis by binding to receptors on target cells [45]. Angiopoietin 2 (Ang-2) is the main factor that promotes angiogenesis. The immune activity of Ang-2 is significantly reduced, which leads to poor development of spiral arteries in the endometrium and affects vasoconstriction. Therefore, in the AA metabolic pathway, PGs may regulate menstruation and haemostasis by upregulating the expression of VEGF and bFGF [46, 47]. TXA2, which is produced by AA metabolism, is a mitogen of vascular smooth muscle cells thereby promoting their proliferation. It also plays a vital role in promoting platelet aggregation [48].

Another AA lipoxygenase family member is 5-lipoxygenase (5-LO), which is encoded by the ALOX5 gene and is a starting catalytic enzyme that bypasses the inflammatory mediators leukotriene (LTs) and lipoxins (LXs). It is widely present in various tissues and blood cells of mammals and has the effect of promoting angiogenesis. Moreover, 5-LO can catalyze the AA biosynthesis of LTs and LXs, whose products have a variety of biological functions and play an important role in the occurrence and development of diseases. AA is converted into 5-hydroperoxyeicosatetraenoic acid (5-HPETE) by 5-LO and 5-lipoxygenase activated protein (5-FLAP). 5-HPETE generates leukotriene A4 (LTA4), leukotriene B4 (LTB4), leukotriene (LTC4), and leukotriene D4 (LTD4) under the catalysis of the enzyme. Among them, LTC4, LTD4 and leukotriene E4 (LTE4) can increase the permeability of capillary venules and promote the contraction of vascular smooth muscles [49].

The caspase-3 protease encoded by the CASP3 gene is the main terminal cleaving enzyme that plays an irreplaceable role in apoptosis. The endometrial functional layer proliferates and exfoliates from the basal layer periodically to form a menstrual cycle, and its mechanism is closely related to apoptosis. Therefore, it is speculated that AA and other compounds in YZG act on the CASP3 gene to treat dysfunctional uterine bleeding through the apoptosis pathway. CASP3 gene balances apoptosis and proliferation, improves the cell cycle, and regulates the normal and cyclic growth, proliferation, and apoptosis of endometrial tissues, which impels the endometrium to shed completely and stops bleeding in time [50].

Beta-sitosterol, a sterol, is a key compound with the highest degree value in YZG. It is found in a wide range of TCMs, such as Cebaiye, Puhuang, Baiji, Danggui, Huaihua. β-Sitosterol inhibits the abnormal proliferation of cells, changes the cell cycle, and plays a role in estrogen regulation, and acts on the hypothalamus-pituitary-ovarian axis and regulates the menstrual cycle. Under normal circumstances, the neuroendocrine mechanism mainly regulates normal menstrual formation through estrogen and progesterone. Studies have shown that the levels of estrogen and progesterone in patients with menstrual dysfunctional uterine bleeding decrease, leading to higher levels of estrogen receptor (ER) and progesterone receptor (PR) expression on the endometrium. PR acts on VEGF, resulting in reduced VEGF secretion and microvascular formation disorders, and leads to abnormal uterine bleeding. β-Sitosterol can play a role in the estrogen effect, bind to the receptor, and regulate the secretion of VEGF. Estrogen can also increase the expression of the cyclic PTGS2 protein in the vaginal and endometrial cells of mice, and PTGS2 is an important rate-limiting enzyme for PGI2. Elevated PTGS2 can inhibit the expression of PGI2. The reduction in PGI2 can reduce the inhibitory effect of platelets and enhance the coagulation effect, thereby achieving hemostasis [51–53].

Leukotriene A4 hydrolase (LTA4H) is a protein that belongs to the peptidase M1 family. LTA4H participates in AA metabolism and is a key enzyme in the AA-mediated inflammatory metabolic network. LTB4 is one of the strongest leucocyte chemokines in the body. It can mediate vascular injury by inducing the release of lysosomal enzymes and reactive oxygen species and participating in systemic lupus erythematosus, skin and kidney tissue inflammation, and tissue damage. Therefore, it is speculated that AA can regulate tissue repair and the inflammatory response by acting on the LTA4H gene, thereby creating a good internal environment in the uterus that is conducive to the shedding and reconstruction of the endometrium [54].

Flavonoids, including skullcapflavone II, salvigenin, and rivularin in YZG can all act on factor VII (FVII) and factor X (FX) receptors. The FVII receptor is considered to be a classic coagulation factor that plays a key role in the coagulation mechanism. When tissues and blood vessels are damaged, tissue factor (TF) is released. TF and FVII or activated FVII (FVIIa) form a complex (TF-FVIIa). This complex can activate FX and factor XI (FXI), and TF released into the blood can significantly promote the coagulation reaction process. Consequently, we can speculate that flavonoids may upregulate F7 and other related genes through the complement and coagulation cascade pathways to increase the release of coagulation factors, enhance coagulation, and accelerate platelet aggregation to achieve hemostasis [55].

There are many flavonoids in YZG that can act on PTGS1 and PTGS2 receptors. It is well known that the PTGS enzymes are related to the production of TXA2. TXA2 is one of the strongest vasoconstrictive and platelet aggregation agents. Platelets can generate TXA2 to make platelets aggregate. PGI2 is an important vasodilator that has a strong antiplatelet aggregation effect. Normally, TXA2 and PGI2 are in a state of dynamic equilibrium in the body. If stimulated by changes in hormone levels, the synthesis of PGI2 increases, and the synthesis of TXA2 decreases, resulting in the inhibition of platelet aggregation and vasodilation, leading to increased bleeding and prolonged menstrual cycles [56, 57]. Animal experimental studies have shown that total flavonoids of Limonium bicolor, Sedum auriculatum, and purple pearl can significantly reduce bleeding and clotting time in mice. The mechanism of action may be through regulating the imbalance of TXA2 and PGI2, enhancing the contraction of small blood vessels in the endometrium to achieve hemostasis [58]. Therefore, we speculate that isorhamnetin, catechins and other flavonoids in YZG also regulate the expression of the PTGS gene to strengthen endometrial vasoconstriction and platelet aggregation, thereby achieving a hemostatic effect.

Although our study basically discussed the molecular mechanisms of YZG, there still exist some limitations. First of all, the data of the study came from existing databases so that the authenticity and integrity of the result rely on the data. Next, the result may not reflect all the real cell network characteristics in the organism. What’s more, further experiments confirming the results of this prediction in YZG are required because our study was performed based on data analysis.

## Conclusions

In the present study, we applied a network pharmacology approach to predict, elucidate, and confirm the potential mechanisms of YZG on ODUB by network construction, integrating target prediction, module analysis, molecular docking and enrichment analysis. The therapeutic efficacy of YZG against ODUB is likely mediated via the regulation of seven targets, namely, PTGS1, PTGS2, ALOX5, CASP3, F7, F10, and LTA4H. In addition, the molecular docking simulation demonstrated a good affinity of PTGS1, PTGS2, ALOX5, CASP3, F7, F10, and LTA4H with their corresponding compounds. GO enrichment analysis illustrated that the targets of YZG against ODUB might be closely associated with the regulation of blood coagulation, regulation of blood coagulation regulation of hemostasis, platelet activation, prostaglandin biosynthetic process, estrogen receptor binding, regulation of heat generation. Additionally, the KEGG pathway enrichment analysis suggested that YZG may simultaneously act on a variety of pathways, including the apoptosis, AA metabolism, serotonergic synapse, complement and coagulation cascade, and C-type lectin receptor signaling pathways. In summary, it can be speculated that the mechanism of YZG in treating ODUB may involve three aspects. First, because the active ingredients regulate endometrial blood vessel contraction, promote platelet aggregation, and induce angiogenesis, YZG is able to promote hemostasis. Second, the active ingredients in YZG exert estrogen and progesterone-like effects that can regulate the menstrual cycle and menstrual flow by acting on the hypothalamus-pituitary-ovary axis. Third, normal menstrual bleeding may be achieved by promoting the apoptosis of the endometrium and causing it to regularly shed. Our results provide a preliminary prediction of the mechanisms related to the therapeutic effect of YZG against ODUB and provide a significant basis and reference for further exploration.

## Data Availability

The datasets used and/or analyzed during the current study are available from the corresponding author upon reasonable request.

## Abbreviations

AA: Arachidonic acid
ALOX5: Arachidonate 5-lipoxygenase
Ang-2: Angiopoietin 2
AR: Androgen Receptor
BDKRB2: Bradykinin receptor B2
bFGF: Basic fibroblast growth factor
CASP: Apoptosis-related cysteine peptidase
CYP3A4: Cytochrome P450 Family 3 Subfamily A Member 4
CXCR: C-X-C Motif Chemokine Receptor
DAVID: Database for annotation, visualization and integrated discovery
DL: Drug likeness
ER: Estrogen receptor
FAS: Factor associated suicide
F7: Coagulation Factor VII
F10: Coagulation Factor X
GABRA: Gamma-Aminobutyric Acid Type A Receptor Subunit Alpha
GABRB2: Gamma-Aminobutyric Acid Type A Receptor Subunit Beta2
GO: Gene Ontology
HSP90AB1: Heat Shock Protein 90 Alpha Family Class B Member 1
KEGG: Kyoto Encyclopedia of Genes and Genomes
LTA4: Leukotriene A4
LTA4H: Leukotriene A4 hydrolase
LTB4: Leukotriene B4
LTC4: Leukotriene C4
LTE4: Leukotriene E4
FVII: Factor VII
FVIIa: Activated FVII
FX: Factor X
LTs: Leukotriene
LXs: Lipoxins
OB: Oral bioavailability
NCOA2: Nuclear Receptor Coactivator 2
ODUB: Ovulatory dysfunctional uterine bleeding
PGE2: Prostaglandin E2
PGF2α: Prostaglandin F2α
PGG2: Prostaglandin G2
PGH2: Prostaglandin H2
PGI2: Prostaglandin I2
PGs: Prostaglandins
PharmGKB: Pharmacogenomics Knowledgebase
PPARG: Peroxisome Proliferator Activated Receptor Gamma
PPARGC1A: Peroxisome Proliferator Activated Receptor Gamma Coactivator 1 Alpha
PPI: Protein–protein interaction
PTGS: Prostaglandin-endoperoxide synthase
PR: Progesterone receptor
RIPK1-STRING: Protein–Protein Interaction Networks Functional Enrichment Analysis
STITCH: Search Tool for Interacting Chemicals
SYK: Spleen Associated Tyrosine Kinase
TCM: Traditional Chinese medicine
TCMSP: Traditional Chinese Medicine Systems Pharmacology Database and Analysis Platform
TF: Tissue factor
TRADD: TNFRSF1A Associated Via Death Domain
TTD: Therapeutic Target Database
TXA2: Thromboxane A2
VEGF: Vascular endothelial growth factor
YZG: Yuzhi Zhixue Granule
5-FLAP: 5-Lipoxygenase activated protein
5-HPETE: 5-Hydroperoxyeicosatetraenoic acid
5-LO: 5-Lipoxygenase
